# A Wolf in Sheep’s Clothing: Reuse of Routinely Obtained Laboratory Data in Research

**DOI:** 10.2196/40516

**Published:** 2022-11-18

**Authors:** L Malin Overmars, Michael S A Niemantsverdriet, T Katrien J Groenhof, Mark C H De Groot, Cornelia A R Hulsbergen-Veelken, Wouter W Van Solinge, Ruben E A Musson, Maarten J Ten Berg, Imo E Hoefer, Saskia Haitjema

**Affiliations:** 1 Central Diagnostic Laboratory University Medical Center Utrecht Utrecht University Utrecht Netherlands; 2 SkylineDx Rotterdam Netherlands; 3 Department of Obstetrics and Gynecology St Antonius Hospital Utrecht Netherlands

**Keywords:** laboratory data, electronic health records, preprocessing, applied data science, laboratory, data, clinical, decision support, decision, research, analysis, patient, value, clinical care

## Abstract

Electronic health records (EHRs) contain valuable data for reuse in science, quality evaluations, and clinical decision support. Because routinely obtained laboratory data are abundantly present, often numeric, generated by certified laboratories, and stored in a structured way, one may assume that they are immediately fit for (re)use in research. However, behind each test result lies an extensive context of choices and considerations, made by both humans and machines, that introduces hidden patterns in the data. If they are unaware, researchers reusing routine laboratory data may eventually draw incorrect conclusions. In this paper, after discussing health care system characteristics on both the macro and micro level, we introduce the reader to hidden aspects of generating structured routine laboratory data in 4 steps (ordering, preanalysis, analysis, and postanalysis) and explain how each of these steps may interfere with the reuse of routine laboratory data. As researchers reusing these data, we underline the importance of domain knowledge of the health care professional, laboratory specialist, data manager, and patient to turn routine laboratory data into meaningful data sets to help obtain relevant insights that create value for clinical care.

## Introduction

The availability of routinely collected laboratory data in electronic health records (EHRs) provides valuable information for medical diagnostics and decision-making in routine care [1]. Data extracted from EHR databases are often reused for deduction of knowledge to perform health care quality evaluations, conduct clinical and epidemiological studies, build clinical decision support systems, and facilitate disease understanding [2]. Moreover, the continuous increase of computing power and the introduction of new machine learning methods will likely further increase the reuse of the vast amounts of routine laboratory data to further personalize care [3]. As laboratory data are abundantly present, often numeric, generated by certified laboratories, and stored in a structured way, one may assume that the data are immediately suitable for (re)use. Technically, this may be true, for such data do not require additional “translation” steps as is the case for unstructured data (eg, written text). However, behind each laboratory test result lies an extensive context of choices and considerations, made by both humans and machines, that introduces hidden patterns in the data. Researchers reusing routine laboratory data, such as epidemiologists and data scientists, who are oblivious to this “world behind the numbers” may either apply or omit (un)necessary preprocessing steps important for the creation of a clinically meaningful data set that may lead to false conclusions. Understanding different steps involved in the generation of routine laboratory data and the insights of domain experts responsible for these steps enables appropriate multidisciplinary preprocessing in medical research.

**Figure 1:**
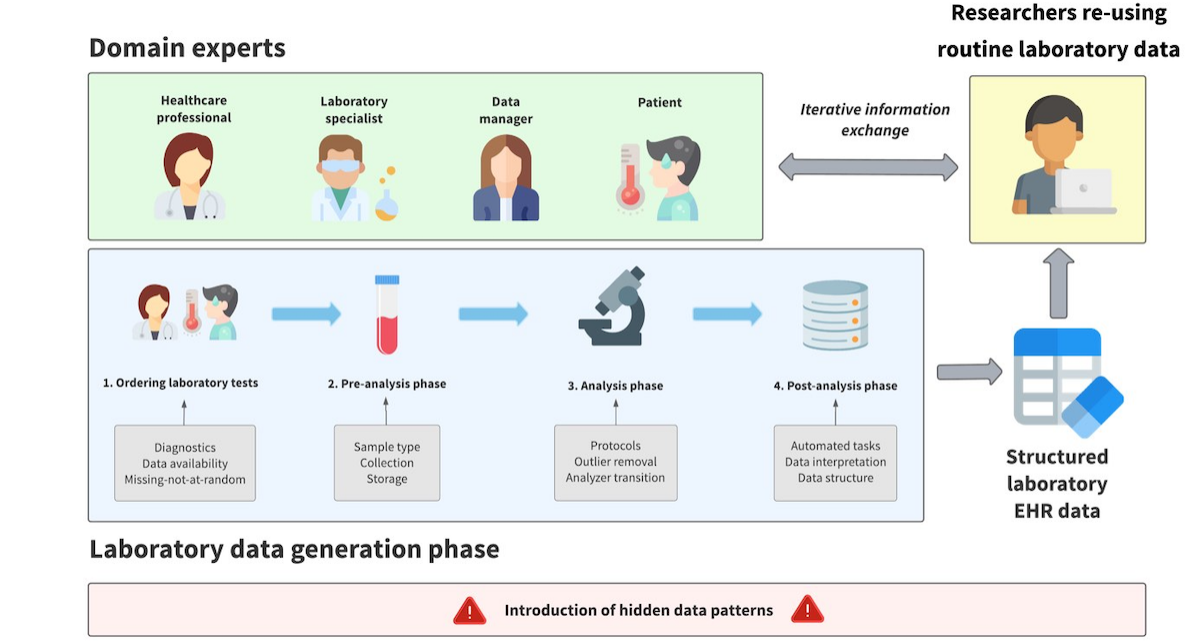
The 4 steps involved in generating structured routine laboratory data and how domain experts and researchers should collaborate in establishing meaningful data sets for reuse.

We will introduce the reader to these hidden aspects of laboratory data originating from the EHR database with examples drawn from our own experience. In 2005, the Central Diagnostic Laboratory of the University Medical Center Utrecht (UMCU) began collecting EHRs, including raw data from our International Organization for Standardization (ISO)-15189 certified laboratory, and compiling them in the Utrecht Patient Orientated Database (UPOD) [4]. With data from the UPOD, our department performed numerous studies in various disciplines over the past 20 years that taught us the necessity of multidisciplinary teams and preprocessing of routine laboratory data to make them suitable for (re)use. We describe the generation of these data in 4 steps and explain how each step may interfere with reuse of the data (Figure 1). First, we provide insights into how laboratory diagnostics is used in clinical care, which largely explains data availability and meaning. To fully understand why a test was ordered in the first place may be difficult (if not impossible) to answer, yet circumstantial evidence can often be found in data patterns and metadata. We also discuss the collection and analysis of specimens from patients in our ISO-15198 controlled laboratory setting. Finally, we elaborate on data processing storage by the laboratory information system (LIS) and EHR database, including automatic calculations and data corrections. We show how every step is reflected in the data, how it can hamper analysis, and then provide possible multidisciplinary preprocessing directions. Increasing the awareness of challenges in preprocessing routine laboratory data and the need for domain expertise may help turn raw laboratory data into meaningful laboratory data that can be reused for translation into value for clinical care.

## Setting the Scene

Before diving into the ordering, (pre)analysis, and storage of routine laboratory data, one needs to be aware of large- and small-scale system characteristics that may affect the data at hand. For example, on a macrolevel, in many health care systems, first-line care is characterized by a more protocolized type of care aimed at the screening and exclusion of severe conditions; therefore, first-line care data carry a high number of “normal” values of a restricted set of laboratory tests. In contrast, specialized physicians in academic hospitals who look for less common diseases may require measurements of more “exotic” parameters, since standard examinations have not yet provided a definite diagnosis, resulting in a high number of abnormal values of a wide range of tests. An interesting development in this regard is the upcoming use of home testing. These tests are performed in uncontrollable collection circumstances and without connection to widely used laboratory information systems, circumventing the auditable data generating, approval, and release procedures we will discuss later in this paper. Limited interoperability between laboratory information systems and home devices may result in duplicate procedures for patients referred to different health care professionals or data storage in different systems that may be available to the treating clinicians but not to the researcher. Moreover, some health care systems reflect a more defensive way of practicing medicine because of medicolegal issues, which means more laboratory data are generated as a result of routine screening procedures, attenuating the meaning of the results.

More specific system characteristics may include the 24/7 availability of resources such as expert laboratory staff, where the absence of laboratory values during the night may simply reflect a closed facility or the other way around—the presence of laboratory values may indicate acute disease during the night and laboratory testing could not wait until the morning. Available metadata in the form of time stamps is indeed collected in the laboratory process and may be used to determine the consecutive steps of the diagnostic workflow. Yet, some of these time stamps outside the lab may be less accurate, as they are written down by hand or indicate preferred collection times. Inside the lab, most time stamps are generated by analyzers and analysis tracks, leading to more precise metadata. Moreover, standard workflow protocols either following international guidelines or local policy may lead to the use of predefined sets of laboratory tests, reflex testing according to specific reference values, or point-of-care testing (POCT), in which the central diagnostic facility is not used due to warranted rapid analysis, preferably near the bedside.

According to the specific research question, patients, physicians, and laboratory specialists can help to provide insights into the local implications of health care systems and indicate workarounds and applicable current and historic clinical and laboratory guidelines that may affect data availability and meaning.

## Step 1. Ordering Laboratory Tests

Ordering laboratory analyses is part of the process in which the patient and the health care professional interact to limit the number of possible differential diagnoses to a minimum. Medical tests meant to confirm or at least discard a potential diagnosis should always be ordered with a clear intent. For instance, a “shotgun approach” (ie, ordering many tests in the hope of being guided by their outcomes) may be a good strategy in the identification of new biomarkers. In modern medicine, however, such an approach will likely only result in high costs with a high chance of spurious findings [5]. The latter is a logical consequence of reference value or reference range definition. In laboratory medicine, reference ranges are defined as the 95% confidence interval. In other words, 95 out of 100 tests of any given parameter will fall within that reference range, and the remaining 5 will be considered an outlier. Statistically, assuming that all parameters to be completely independently regulated, testing for 5 parameters would result in 23% chance of at least 1 outlier, testing for 10 would result in a 40% chance, and testing for 20 would result in a 64% chance. While multiple comparisons in studies can be managed by applying correction factors, this is not done in clinical practice. Fortunately, diagnostic means such as laboratory tests are ordered with this knowledge in mind. Conversely, *not* testing for a specific parameter also needs to be regarded as meaningful, as the said parameter was most likely not considered to contribute to clinical decision-making. This is referred to as missing not at random (MNAR) patterns (ie, the choice for or against a test itself already contains meaning) [6].

In other words, many factors affect data availability. This has major implications for handling missing values. In general, it is not recommended to carelessly impute routine laboratory data, given the many different possible underlying reasons for missingness (Table 1). Unfortunately, the preceding deliberations that climax in the test order are usually not captured by order forms, in contrast to radiology or pathology requests, where the clinical question is an integral part (“pneumonia?” or “metastasis?”) [7]. A single laboratory test order can also be used to rule out or confirm more than 1 clinical question. Accordingly, the interpretation of a laboratory test result may differ between different clinical questions so that an identical result can have completely different meanings. This may be obvious in many cases. For example, a normal hemoglobin level in an elite athlete can be expected. In a patient with chronic fatigue, this may mean that the fatigue is probably not caused by anemia, whereas a normal hemoglobin level in a polytrauma patient with severe blood loss most likely only reflects successful blood transfusions. To make it more complicated, the interpretation of laboratory test results depends on the acuteness of the disease. For example, acute versus chronic anemia can acutely result from subacute severe hemorrhage but can also follow chronic blood loss in small amounts, iron deficiency, or bone marrow suppression.

The process of clinical reasoning is not part of laboratory standard operating procedures, so unfortunately, from a reuse point of view, it is not captured in any (meta)data. Because it is influenced by multiple factors, it can introduce patterns in data. Therefore, clinical characteristics influence availability and meaning in this very first step of data generation and are among the hardest to discover and, consequently, the hardest to model in projects reusing laboratory data. Physicians and patients as domain experts may help shed some light in this step, for example on how to distinguish between the different meanings of missing data (see Table 1 as a conversation starter), yet the specific choices that affect data availability may vary.

**Table 1:** Examples of physician-initiated and system-initiated processes that may affect the presence of laboratory values.

Reasons (not) to perform laboratory diagnostics			Example		Meaningful missingness pattern
**Physician-initiated**					
	No laboratory measurements needed because there is no sign the of the disease	No CRP^a^ measurement was ordered because patient did not have a fever		CRP missing: most likely means a *normal* value	
	No laboratory measurements needed because the disease is very obvious	No herpes zoster antibodies were ordered because the patient displayed shingles		Antibodies missing: most likely means an *abnormal* value	
	To confirm a diagnosis	Hemoglobin ordered for anemia		Hemoglobin missing: may either mean *normal* value or *abnormal* value	
	To exclude diagnosis	Metanephrines ordered for pheochromocytoma		Metanephrines missing: may either mean *normal* value or *abnormal* value	
	To determine treatment	Assess kidney function to titrate dosage of antibiotics		Kidney function missing: probably means a *normal* value.	
	Preprocedural risk assessment	Determination of INR^b^ before thrombolysis		INR missing: probably means a *normal* value.	
	To monitor disease activity	Bacterial infection followed up by a lactate measurement		Lactate missing: most likely means a *normal* value	
	To monitor treatment effect	CA125 ovarian tumor marker ordered		CA125 missing: most likely means a *normal* value	
	Drug adherence	Low density lipoprotein cholesterol ordered		LDL^c^ missing: probably means a *normal* value	
	Physician uncertainty or the reassurance of a patient	Lyme disease screening ordered		Lyme disease screening missing: most likely means a *normal* value	
	Screening	Fecal occult blood test for colorectal cancer		Fecal occult blood test missing: probably means a *normal* value.	
	Periodic health check-ups	Fasting glucose test for diabetes		Fasting glucose missing: most likely means a *normal* value	
**System-initiated**					
	Standard protocol within EHR^d^	Specific triaging protocol in the emergency department for suspected sepsis		Missingness describes the patient population: sepsis not suspected	
	Standard combinations of parameters that cannot be ordered separately	Hemoglobin and hematocrit		Missingness of either may indicate the measurement has failed	
	Reflex tests	Free T4 is measured only when TSH^e^ is outside reference range		Missingness of free T4 if TSH is available means that free T4 is most likely *normal*	
	Automatic alerts in laboratory analyzers	Immature cells detected in blood by hematology analyzer		Missingness of immature blood cells means there are none detected	

^a^CRP: c-reactive protein.

^b^INR: international normalized ratio.

^c^LDL: low-density lipoprotein.

^d^EHR: electronic health record.

^e^TSH: thyroid-stimulating hormone.

## Step 2. Before the Laboratory: Preanalysis Phase

Depending on the considerations by the health care professional and their patient, the clinical presentation, and the ordered test, the next choice to be made concerns the collection of biological material. Blood and urine collection are widely known, but modern laboratories run diagnostic tests on many more body fluids and materials, such as spinal fluid, serosal fluids, or feces. The number of possibilities further increases by (1) the method of sample collection (eg, venous vs capillary blood sampling) and (2) the collection material (eg, ethylenediaminetetraacetic acid vs citrate-buffered blood collection tubes). These 2 factors may seem minor at first glance but have a major impact on the “test menu” (ie, which analyses can be performed as well as result reliability). For example, in the UMCU, there are over 2900 different tests. Some tests can only be performed using specific collection tubes, and while some can be performed in minute amounts, others may require sample volumes that preclude capillary blood sampling. Finally, time is relevant. For example, glucose levels in whole blood samples significantly decrease over time due to their consumption by blood cells [8]—not to mention circadian variations of numerous analytes. Laboratory specialists are trained to support the treating physician in assessing the possibilities and making the right decision to avoid diagnostic and treatment delay, unnecessary additional testing, and associated costs. Altogether, this phase is usually referred to as “preanalysis.”

The following example illustrates the often-underestimated impact of the preanalytic choices: A “simple” glucose level can be measured in various body fluids, such as blood or liquor (spinal fluid) in this case. Glucose levels in liquor are usually 30% to 40% lower than in blood [9]. Another well-known example is the concentration difference of creatinine in blood and urine. On an individual level, such factors may be easy to look up and adjudicate. In large data sets, this becomes impossible. A glucose level of 50 mg/dL may mean that it was measured in liquor and can be considered “normal,” whereas when measured in blood, it may indicate a hypoglycemia. Therefore, is it essential to account for specimen types in available metadata and perform the preprocessing accordingly by either filtering out measurements or annotating them as separate variables to avoid false interpretation. Standards for laboratory test identification and naming have been proposed but are unfortunately not widely adopted [10]. Hence, laboratory specialists and data managers must cooperate when interpreting available data sources and deduce specimen type in the absence of unique identifiers that account for the available combinations of the aforementioned factors.

In addition to intrinsic circadian variations, patient behavior and actions before and/or during specimen collection (eg, medication or food intake) can affect test results. A well-known example is the variability of blood glucose levels in relation to recent food intake. Therefore, blood glucose concentrations are usually measured after at least 8 hours of fasting. In practice, not all patients faithfully report previous eating or drinking (other than water, unsweetened tea, or coffee) before sample collection. Therefore, even controlled and lege artis performed sample collection and analysis cannot guarantee a valid reflection of patient status. As such, accurate annotation (fasting/nonfasting), where possible, is important to either remove or include these measurements depending on the research question. Patient compliance to drugs or instructions varies significantly between treatments or patient groups. Physicians can help identify or even point to specific patients or patient groups that might distort the data set, thereby providing additional meaning to the data.

## Step 3. In the Laboratory: Analysis Phase

Clinical laboratories use different types of analyzers to perform tests on the obtained sample. New analyzers, reagents, biomarkers, and software packages are introduced and updated frequently, and tests can be added, removed, or changed. Analyzers are regularly calibrated, but both distribution shifts and continuity imperfections within calibration ranges may occur over time and become apparent in large data sets. Although in most cases analytes are named identically across different laboratories, their actual level may differ significantly even when the same sample is measured. This can be due to different types of analyzers, different assay types, or different analyzer/assay manufacturers [11]. It is critical to note that the resulting differences in reported analyte concentrations can be substantial when comparing assays from different providers. In a nutshell, analyte X from provider Y is not the same as analyte X from provider Z. Harmonization efforts are ongoing but not yet widely implemented [12].

Within a given laboratory, the usual lifespan of analyzers is around 10 years. In other words, even in 1 hospital, analyzers need to be replaced every now and then. Accordingly, after transition, reported analyte concentrations can change from one day to the next. In certified laboratories, transition between analyzers is accompanied by an intermediate calibration phase in which the new analyzer is aligned. These calibration data are generated on specific test samples and do not usually appear in clinical data. However, when data are analyzed over a longer period, the analyzer transition can still be visible in the data. As an example, in 2013, our laboratory recalibrated the serum creatinine analysis; as a result, mean serum creatinine values were lower in the following years. Not having this background knowledge could easily result in the misinterpretation laboratory values over time and is therefore an essential step in quality control. Metadata analysis can help with identifying such changes and performing adequate correction actions.

Population-specific reference ranges (eg, age or sex dependent) are a form of clinical decision support, as they guide the physician in interpreting test results in clinical practice. Researchers reusing routine laboratory data can use these reference ranges to establish whether a test result deviates from a healthy population, assuming said reference indeed reflects health status. Reference ranges correspond to a set of values covering 95% of the results from testing a reference, such as a healthy population. Such populations are carefully selected in the approval process of any assay and analyzer based on defined criteria, such as sex, age, and pregnancy. These ranges are usually known along with the test result but may differ between laboratories, limiting the interoperability of findings. Grossly increased values that significantly deviate from reference values (eg, creatinine kinase in myocardial infarction and human chorionic gonadotropin in pregnancy) may seem outliers from a statistical point of view, but they may actually reflect the state of the human body and need to be interpreted on a log scale, as they are almost never the result of analytical error.

Initial outlier detection takes place in the laboratory as part of quality assurance and validation, where measurement errors are typically identified, signified, and even corrected. Therefore, applying rigorous outlier detection and removal by data reusers may remove highly informative test results and should be carefully deployed. Moreover, reference ranges can be modified over time. This can be the case when analyzers or assays change, as mentioned earlier in this paper, but can also be the case when new evidence becomes available. In cardiovascular medicine, for example, sex-specific reference thresholds are currently applied for cardiac troponin levels, a marker of cardiomyocyte damage or injury [13].

The LIS is the interface between analyzers and the EHR database. In addition to its role in test order management, it performs a variety of automated tasks essential for routine clinical care. It plays a central role in identifying aberrant measurements and performing simple and complex calculations such as low-density lipoprotein (LDL) cholesterol calculation with the Friedewald formula [14]. These alerts and formulas may change over time, potentially resulting in inconsistent data or breaks in trends. Kidney function is usually monitored using an estimation of the glomerular filtration rate (GFR) instead of performing a “real” measurement, including urine collection, for 24 hours. Several formulas have been established and are applied clinically to this end. Our laboratory replaced the Modification of Diet in Renal Disease (MDRD) with the Chronic Kidney Disease Epidemiology Collaboration (CDK-EPI) formula in 2013. Consequently, the distribution of GFR measurements changed significantly over time due to considerable variations between the resulting minimum and maximum results. Depending on the research question at hand, such ambiguities may be resolved by recomputing the GFR for all patients with the same formula to obtain a single distribution when studying physiological processes. However, when studying health care processes, GFR values should not be recomputed, as health care professionals were only provided with the value calculated at that time and made their decisions accordingly. Thus, the choice whether using reported or recomputed data largely depends on the research question.

Laboratory values are only released into the LIS and EHR database after they have been approved by a laboratory specialist or a prespecified protocol. Values that surpass the preset assay specification (ie, lower limit of detection and upper limit of quantification) may be reported as “less than” or “greater than,” respectively, and these modifiers may be found as a text variable in a different data location. The “greater than” and ”less than” signs mean that the results fall outside the measurement range of the assay. However, this does not mean that they are incorrect, as laboratory specialists review these values and check their plausibility before approving them for reporting in the EHR. Some analyses may be of such complexity that they cannot be done by an automated analyzer and thus require manual interpretation by a laboratory specialist before release (eg, blood smears, bone marrow morphology, or specific blood type analyses). These are often accompanied by a free text comment from the laboratory specialist to aid the clinician with interpretation and may require natural language processing before being used further.

## Step 4. After the Laboratory: Postanalysis Phase

After having been checked and approved, test results are reported in the LIS together with metadata describing analyzer configurations, sampling time, assay time, and more. The clinical laboratory is responsible for the correct laboratory value provision in clinical practice. However, for some analytes, only the result of the test ordered by the physician, accompanying comments of the laboratory specialist are eventually stored in the EHR, and raw data and metadata may be only available in the LIS. For specific research questions, such metadata can be indispensable for correct data analysis and interpretation and should therefore be retrieved in collaboration between data managers and laboratory specialists.

As previously exemplified in this paper, data continuity can be infringed at various levels. Analyzer updates may lead to different parameter names. Switching to a different analyzer, LIS, or EHR system can cause significant data variation. A parameter name change may seem minor for a human, like the addition of a single letter. In database terms, this may mean that a data column is added at a point in time, while no new data are added to the column reflecting the “old” name. This can be overcome with specialist knowledge input to merge data before and after the name was changed, provided that the change was indeed limited to the name and that the distribution of results has not changed over time. Doing so leads to a strong reduction in missing data. A variable name change can also be accompanied by a change in the definition of that parameter, resulting in aberrant distributions. Quantile-quantile and density plots can be used to demonstrate shifts after which they can be substantiated with, for example, a Kolmogorov-Smirnov test. Laboratory specialists are needed to help pointing out the cause of the change and help decide on preprocessing protocols and if data can be merged. In the future, data standards such as Logical Observation Identifiers Names and Codes (LOINC) or Systematized Nomenclature of Medicine Clinical Terms (SNOMED) may partly resolve some of these issues.

## Conclusion

Routine laboratory data are a potential information gold mine for reuse beyond their purpose for care, as they are abundantly present, most often numeric, generated by certified laboratories, and stored in a structured manner. However, they can be a wolf in sheep’s clothing. To fully exploit their potential, one should be aware of their caveats because statistical methods are generally not designed to incorporate their intricate clinical context, particularly not in the absence of metadata. Laboratory values are only part of the full clinical picture that health care professionals use to diagnose and treat patients. Moreover, this picture is merely reflected and not fully captured by routine health care data. Therefore, as researchers reusing routine laboratory data, we underline the prerequisite of collaboration between the health care professional, laboratory specialist, data manager, and patient to accurately recreate a valuable proxy of the clinical state of the patient by addressing and resolving all imperfections in electronic health record data, especially laboratory data [15]. Instead of massive computing power, this collaborative human effort may well be the only way to leverage the potential of artificial intelligence by turning routine data into meaningful data sets to help obtain relevant insights that create value for clinical care.

## Abbreviations

CDK-EPI: Chronic Kidney Disease Epidemiology Collaboration
EHR: electronic health record
GFR: glomerular filtration rate
ISO: International Organization for Standardization
LDL: low-density lipoprotein
LIS: laboratory information system
LOINC: Logical Observation Identifiers Names and Codes
MDRD: Modification of Diet in Renal Disease
MNAR: missing not at random
POCT: point-of-care testing
SNOMED: Systematized Nomenclature of Medicine Clinical Terms
UMCU: University Medical Center Utrecht
UPOD: Utrecht Patient Orientated Database

## References

[1] Blumenthal D, Glaser JP (2007). Information technology comes to medicine. N Engl J Med.

[2] Meystre SM, Lovis C, Bürkle T, Tognola G, Budrionis A, Lehmann CU (2017). Clinical data reuse or secondary use: current status and potential future progress. Yearb Med Inform.

[3] Triantafyllidis AK, Tsanas A (2019). Applications of machine learning in real-life digital health interventions: review of the literature. J Med Internet Res.

[4] Ten Berg BM, Huisman A, Van den Bemt PMLA, Schobben AFAM, Egberts ACG, Van Solinge WW (2007). Linking laboratory and medication data: New opportunities for pharmacoepidemiological research. CCLM.

[5] Bindraban RS, van Beneden ML, Kramer MH, van Solinge WW, Neppelenbroek SI, van Wijnen M, Griffioen-Keijzer A, Al-Dulaimy M, Ten Berg MJ, Nanayakkara PW (2018). A multicenter before-after study on reducing unnecessary diagnostics by changing the attitude of caregivers: protocol for the RODEO Project. JMIR Res Protoc.

[6] Groenwold RHH (2020). Informative missingness in electronic health record systems: the curse of knowing. Diagn Progn Res.

[7] Agniel D, Kohane IS, Weber GM (2018). Biases in electronic health record data due to processes within the healthcare system: retrospective observational study. BMJ.

[8] Fischer M, Hannemann A, Winter T, Schäfer C, Petersmann A, Nauck M (2021). Relative efficacy of different strategies for inhibition of in vitro glycolysis. Clin Chem.

[9] Mundt L (2015). Graff’s Textbook of Urinalysis and Body Fluids.

[10] Bodenreider O, Cornet R, Vreeman D (2018). Recent Developments in Clinical Terminologies - SNOMED CT, LOINC, and RxNorm. Yearb Med Inform.

[11] Allardet-Servent J, Lebsir M, Dubroca C, Fabrigoule M, Jordana S, Signouret T, Castanier M, Thomas G, Soundaravelou R, Lepidi A, Delapierre L, Penaranda G, Halfon P, Seghboyan J (2017). Point-of-care versus central laboratory measurements of hemoglobin, hematocrit, glucose, bicarbonate and electrolytes: a prospective observational study in critically ill patients. PLoS One.

[12] Plebani M (2018). Harmonization in laboratory medicine: more than clinical chemistry?. Clin Chem Lab Med.

[13] Lee KK, Ferry AV, Anand A, Strachan FE, Chapman AR, Kimenai DM, Meex SJR, Berry C, Findlay I, Reid A, Cruickshank A, Gray A, Collinson PO, Apple FS, McAllister DA, Maguire D, Fox KAA, Newby DE, Tuck C, Keerie C, Weir CJ, Shah ASV, Mills NL, High-STEACS Investigators (2019). Sex-specific thresholds of high-sensitivity troponin in patients with suspected acute coronary syndrome. J Am Coll Cardiol.

[14] Friedewald W, Levy R, Fredrickson D (1972). Estimation of the concentration of low-density lipoprotein cholesterol in plasma, without use of the preparative ultracentrifuge. Clin Chem.

[15] Bezemer T, de Groot MC, Blasse E, Ten Berg MJ, Kappen TH, Bredenoord AL, van Solinge WW, Hoefer IE, Haitjema S (2019). A human(e) factor in clinical decision support systems. J Med Internet Res.

